# Targeted Drug Delivery Biopolymers Effectively Inhibit Breast Tumor Growth and Prevent Doxorubicin-Induced Cardiotoxicity

**DOI:** 10.3390/molecules27113371

**Published:** 2022-05-24

**Authors:** Sonja Dragojevic, Jung Su Ryu, Michael E. Hall, Drazen Raucher

**Affiliations:** 1 Division of Radiation Oncology, Mayo Clinic and Foundation, 200 First Street, Rochester, MN 55905, USA; dragojevic.sonja@mayo.edu; 2 Department of Cell and Molecular Biology, University of Mississippi Medical Center, 2500 North State Street, Jackson, MS 39216, USA; jryu@umc.edu; 3 Department of Medicine, University of Mississippi Medical Center, 2500 North State Street, Jackson, MS 39216, USA; mehall@umc.edu

**Keywords:** elastin-like polypeptide, drug delivery, breast cancer, doxorubicin, cardiotoxicity

## Abstract

The anticancer agent doxorubicin(dox) has been widely used in the treatment of a variety of hematological malignancies and solid tumors. Despite doxorubicin’s efficiency in killing tumor cells, severe damage to healthy tissues, along with cardiotoxicity, limits its clinical use. To overcome these adverse side effects, improve patient safety, and enhance therapeutic efficacy, we have designed a thermally responsive biopolymer doxorubicin carrier that can be specifically targeted to tumor tissue by locally applying mild hyperthermia (41 °C). The developed drug vehicle is composed of the following: a cell penetrating peptide (SynB1) to promote tumor and cellular uptake; thermally responsive Elastin-like polypeptide (ELP); and the (6-maleimidocaproyl) hydrazone derivative of doxorubicin (DOXO-EMCH) containing a pH-sensitive hydrazone linker that releases doxorubicin in the acidic tumor environment. We used the in vivo imaging system, IVIS, to determine biodistribution of doxorubicin-delivered ELP in MDA-MB-231 xenografts in nude mice. Tumor bearing mice were treated with a single IV injection of 10 mg/kg doxorubicin equivalent dose with free doxorubicin, thermally responsive SynB1 ELP 1-DOXO, and a thermally nonresponsive control biopolymer, SynB1 ELP 2-DOXO. Following a 2 h treatment with hyperthermia, tumors showed a 2-fold higher uptake when treated with SynB1 ELP 1-DOXO compared to free doxorubicin. Accumulation of the thermally non-responsive control SynB1 ELP2 –DOXO was comparable to free doxorubicin, indicating that an increase in dox accumulation with ELP is due to aggregation in response to thermal targeting. Higher levels of SynB1 ELP1–DOXO and SynB1 ELP2 –DOXO with respect to free doxorubicin were observed in kidneys. Fluorescence intensity from hearts of animals treated with SynB1 ELP1–DOXO show a 5-fold decrease in accumulation of doxorubicin than the same dose of free doxorubicin. SynB1-ELP1-DOXO biopolymers demonstrated a 6-fold increase in tumor/heart ratio in comparison to free doxorubicin, indicating preferential accumulation of the drug in tumors. These results demonstrate that thermally targeted polymers are a promising therapy to enhance tumor targeting and uptake of anticancer drugs and to minimize free drug toxicity in healthy tissues, representing a great potential for clinical application.

## 1. Introduction

Breast cancer is the most common cancer affecting women across the world, with prevalence and mortality rates expected to increase considerably in the next years [1,2]. Current treatment approach is multimodal and often includes mastectomy, followed by concurrent radio- and chemotherapy [3]. This standard therapy approach has been used for decades in breast cancer treatment; however, the outcomes for patients are inadequate, and often result in incomplete cancer remission or cancer recurrence.

Many widely used chemotherapeutics in the treatment of breast cancer are small molecule drugs which distribute through the whole body, affecting not only the cancerous cells, but also healthy tissues. One such chemotherapeutic is the commonly used anticancer agent doxorubicin (dox), which has extensive application in treatment of a variety of hematological malignancies and solid tumors [4,5]. Despite dox’s efficiency in eradicating tumor cells, severe damage to healthy tissues, along with irreversible, dose-dependent damage to cardiac muscle tissue, limits its use in clinics [6].

Therefore, the imperative to work within the narrow balance of a Dox dose sufficient to effectively treat cancer and one likely to cause permanent cardiac damage creates a clinical dilemma as daunting as it is routine. This daily challenge lends urgency to the need to discover and advance delivery systems able to selectively target the delivery of anticancer drugs to tumor sites so as to avoid off-target effects, particularly cardiac damage. Current approaches in alleviating the detrimental side effects of doxorubicin were designed as a different formulation of macromolecules, liposomes, hydrogels, and nanoparticles in order to improve overall dox delivery and abate cardiac toxicity [7,8,9,10]. However, these approaches have limited success in clinics, and therefore novel drug delivery approaches are urgently needed. To address this need, and to overcome adverse side effects, improve patient safety, and enhance therapeutic efficacy, we have designed a thermally responsive biopolymer dox carrier that can be specifically targeted to tumor tissue by locally applying mild hyperthermia (41 °C). The developed drug vehicle is composed of a cell penetrating peptide (SynB1) to promote tumor and cellular uptake; thermally responsive elastin-like polypeptide (ELP); and the (6-maleimidocaproyl) hydrazone derivative of doxorubicin (DOXO-EMCH) containing a pH-sensitive hydrazone linker that releases dox in the acidic tumor environment. Hyperthermia enhances preferential accumulation of ELP delivered DOXO in tumors. The cell penetrating peptide, SynB1, mediates uptake of ELP delivered DOXO in tumor tissues and cancer cells.

We have previously shown that thermoresponsive biopolymer SynB1-ELP-GGC can be used in delivery of small molecule chemodrugs in vitro in MCF-7 breast cancer cell line [11,12]. SynB1-ELP1-(DOXO)3 in vitro efficacy was later expanded and in vivo experiments were conducted in E0771 murine breast cancer in C57BL/6 mice [13]. Here we expand and complement those studies using a more clinically relevant model, MDA MB 231 human breast cancer xenografts in female athymic nu/nu mice. The study objectives were: (i) to develop an externally triggered drug delivery system to selectively deliver chemo-drugs to breast tumors and increase treatment effectiveness and reduce side effects; (ii) quantitate the in vivo distribution of CPP-ELP-Dox, CPP-ELP, ELP, and free Dox in normal and neoplastic tissue in a tumor-bearing xenograft mouse model of breast cancer; (iii) evaluate therapeutic biopolymer drug conjugate efficacy, with and without hyperthermia, in the murine xenograft models using human breast cancer cell line MDA-MB-231 triple negative (ER-, Her2-, PR-) and assess toxicity. Our results show that dox can be specifically targeted to the tumor and effectively inhibit tumor growth, while alleviating side effects, specifically decreased accumulation in heart tissue.

In summary, developed externally triggered drug delivery system confers selective delivery of anti-cancer drugs to breast tumors by its unique features: (1) the passive targeting properties of macromolecular carriers that derives from the enhanced permeability and retention effect, (2) active drug targeting to tumor sites by a clinically available external trigger (mild hyperthermia), and (3) efficient, intracellular tumor drug delivery mediated by a cell penetrating peptide to reduce tumor growth, improve treatment outcomes, and retain better patient quality of life.

## 2. Results

### 2.1. Design of ELP Based Doxorubicin Carrier

**Figure 1:**
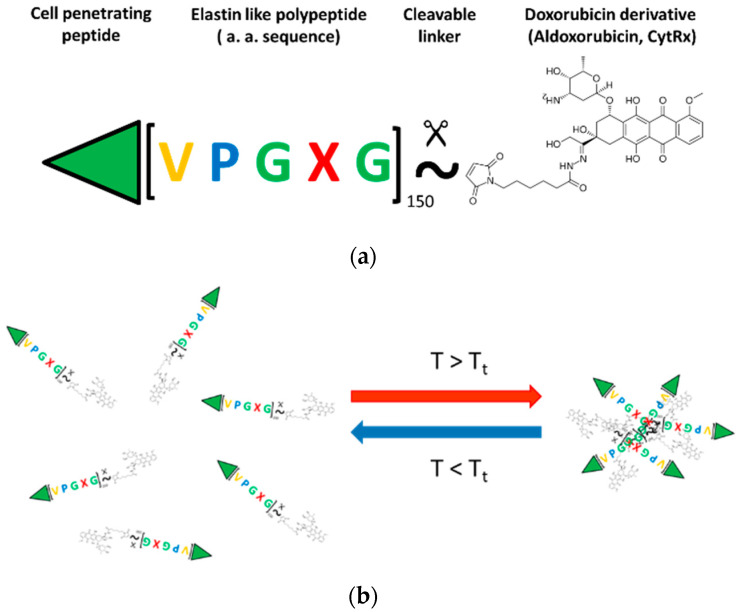
Schematics of the ELP-based drug delivery vector. (**a**) The developed drug vehicle is composed of a cell penetrating peptide (SynB1) to promote tumor and cellular uptake; thermally responsive elastin-like polypeptide (ELP); and the (6-maleimidocaproyl) hydrazone derivative of doxorubicin (DOXO-EMCH) containing a pH-sensitive hydrazine linker that releases doxorubicin derivative in the acidic tumor environment. (**b**) Below transition temperature ELP is present in the solution as a monomer. When temperature is increased above transition temperature ELP macromolecules form aggregates.

To increase treatment effectiveness and reduce side effects, we have developed an externally triggered drug delivery system to selectively deliver dox to breast cancer tumor tissue. This drug carrier relies on the thermally responsive biopolymer, the elastin-like polypeptide (ELP), which is soluble at physiological temperatures (37 °C), but undergoes a phase transition and aggregates at an externally applied mild hyperthermia at 40–41 °C (Figure 1). The system allows targeted delivery to spare healthy tissues. The ELP-Dox conjugate consists of a cell penetrating peptide (CPP) at the N-terminus and the 6-maleimidocaproyl hydrazone derivative of doxorubicin at the C-terminus of the ELP. An acid-sensitive hydrazone linker ensures DOX’s release in the lysosomes/endosomes after cellular uptake of the drug conjugate [14,15,16,17].

### 2.2. Characterization of Transition Temperature of Biopolymer-Drug Conjugates

**Figure 2:**
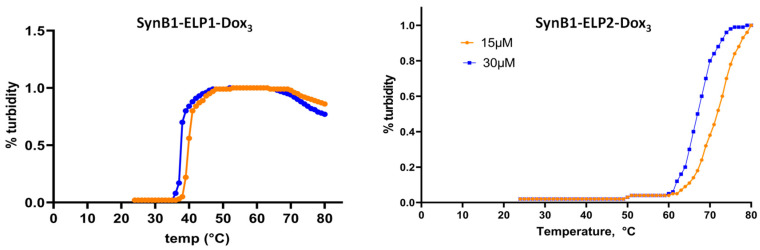
Phase transition of the proteins as a function of temperature. The aggregation of each protein with varying temperature was measured by the turbidity of protein solution heated at a rate of 1 °C/per min and measuring absorbance at 350 nm. Rapid increases in absorbance were observed around the transition temperature of each protein.

In order to determine transition temperature of SynB1-ELP1- DOXO, and SynB1-ELP2-DOXO we evaluated the phase transition of the ELP constructs as a function of temperature by measuring turbidity (Figure 2).

Figure 2 shows that thermoresponsive SynB1-ELP1-DOXO is present as a monomer, and therefore not causing any turbidity, at the temperatures below 38 degrees C. However, when temperature is raised, there is a sharp increase in solution turbidity, indicating ELP phase transition and formation of macromolecular particles. Similarly, non-themoresponsive control SynB1-ELP2-DOXO, undergoes phase transition above 60 degrees C (Figure 2). Thus, in this experiment we confirmed that all ELP constructs showed thermoresponsiveness to changes in ambient temperature.

### 2.3. Biodistribution of Biopolymer-Drug Conjugates vs. Free Dox

**Figure 3:**
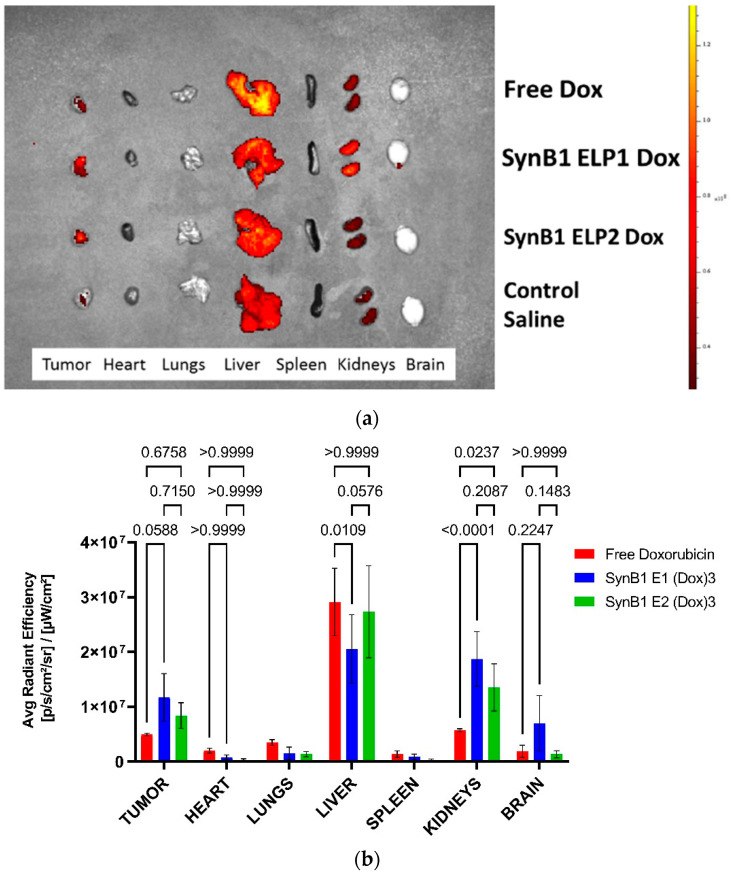
Biodistribution of dox in MDA MB 231 tumor-bearing mice. (**a**) Representative ex vivo fluorescent images of DOX signal accumulated in tumors and organs following intravenous injection of mice with free dox, SynB1-ELP1-DOXO and thermally non-responsive control SynB1-ELP2-DOXO, (**b**) Quantification of dox fluorescent signals in region of interests (ROIs) for tumor and organs, 2 h after treatment.

To determine the biodistribution of dox-delivered ELP in MDA-MB231 triple negative human breast cancer cell line xenografts in nude mice, we used the in vivo imaging system, IVIS. Animals were treated with 10 mg/kg of free dox, SynB1-ELP1-DOXO, SynB1-ELP2-DOXO, and saline. After 2-h treatment with hyperthermia, as previously described [18], mice were sacrificed and organs were harvested and the excised organs were examined using ex vivo fluorescence imaging (Figure 3a).

Following a 2-h treatment with hyperthermia, tumors showed a 2-fold higher uptake when treated with SynB1-ELP1-DOXO compared to free dox. Accumulation of the thermally non-responsive control SynB1-ELP2-DOXO was less than SynB1-ELP1-DOXO, indicating that an increase in dox accumulation with ELP is due to aggregation in response to thermal targeting. Two to three-fold higher levels of SynB1-ELP1-DOXO and SynB1-ELP2-DOXO with respect to free doxorubicin were observed in kidneys. 

### 2.4. Biopolymer-Delivered Dox Accumulates More in the Tumor but Less in the Heart Compared to Free Dox

**Figure 4:**
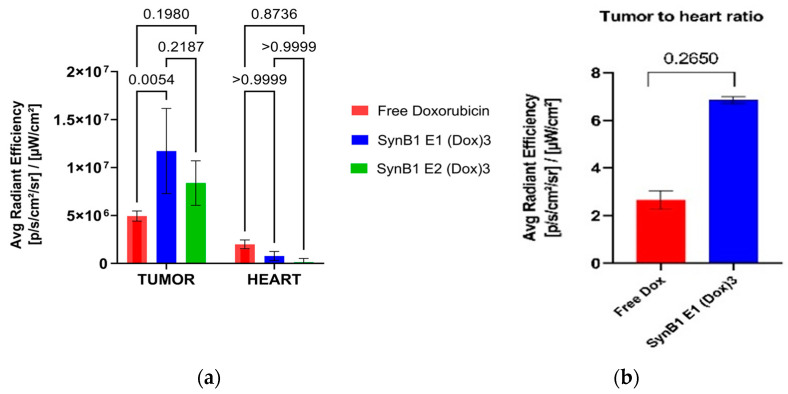
Dox accumulation in heart and tumor. Tumor-bearing nude mice were treated with either 10 mg Dox equivalent/kg free dox or biopolymer-dox in the absence or presence of hyperthermia. (**a**) Dox fluorescence intensity in tumor and heart samples of treated animals. (**b**) Ratio of tumor to heart dox concentration at 6 h post-injection. *p* < 0.01 (ANOVA, Bonferroni contrast, mean ± s.d.; *n* = 5).

To evaluate whether or not dox tumor levels are enhanced by the thermal targeting of biopolymer-dox as compared to free dox, tumor samples were analyzed for dox fluorescence. A more than 2-fold increase in dox tumor-uptake levels was observed after biopolymer-dox treatment in combination with hyperthermia, versus treatment with only free drug (Figure 3a). In addition, as dox accumulation in heart tissue poses a major concern, we assayed heart samples from treated animals. Fluorescence intensity from hearts of animals treated with SynB1-ELP1-DOXO show a 5-fold lower accumulation of doxorubicin than the same dose of free doxorubicin (Figure 4a). SynB1-ELP1-DOXO biopolymers demonstrated a 6-fold increase in tumor/heart ratio (Figure 4b) in comparison to free doxorubicin, indicating preferential accumulation of the drug carrier in tumors. As dox-related cardiotoxicity is dose-limiting, this observation may have meaningful implications for the therapeutic use of biopolymer-dox. These results indicate that with thermal targeting, biopolymer-dox can potentially reduce cardiotoxicity by steering dox away from the heart and targeting it to the tumor.

### 2.5. Biopolymer-Delivered Dox Does Not Affect Cardiac Function as Free Dox

**Figure 5:**
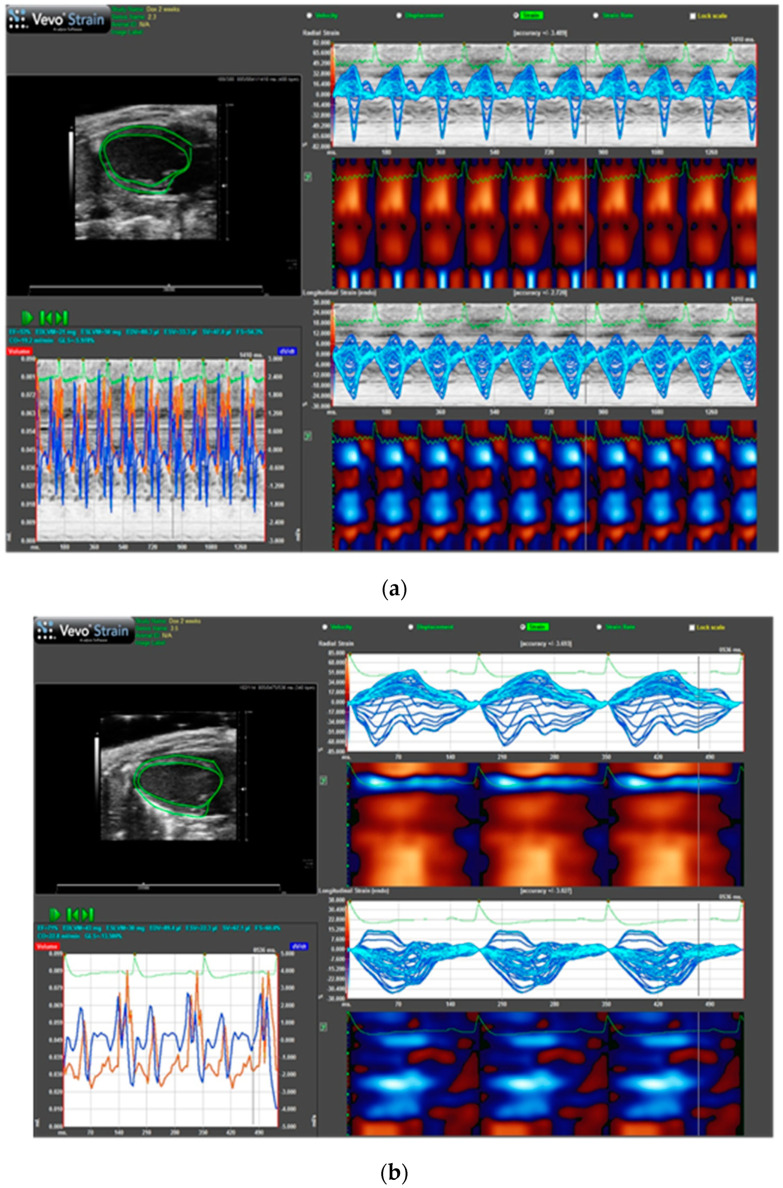
Speckle-tracking echo (strain). Animals were treated on days 1, 4 and 7 with the same dose of (**a**) free doxorubicin or (**b**) SynB1-ELP1-DOXO, and echocardiography was performed two weeks after the last treatment.

To compare cardiac function between free Dox and SynB1-ELP1-DOXO treated animals we performed echocardiography. The parameters of myocardial deformation, including the strain rate derived from strain analysis, provide valuable information for detecting early myocardial abnormalities following dox treatment. Figure 5 shows speckle-tracking echo (strain) demonstrating reduced ejection fraction (53% vs. 71%) and impaired global longitudinal strain (−5.9% vs. −13.5%) in mice treated with 7.5 mg/kg dox (Figure 5a) compared to mice treated with the same concentration of biopolymer-dox (Figure 5b). The results demonstrate that the cardiotoxicity of dox seriously impaired cardiac function. In comparison, after treatment with biopolymer-dox, minimal differences when compared with the control group were observed (data not shown).

### 2.6. Intratumoral Distribution of Free Drug and SynB1-ELP-DOXO

**Figure 6:**
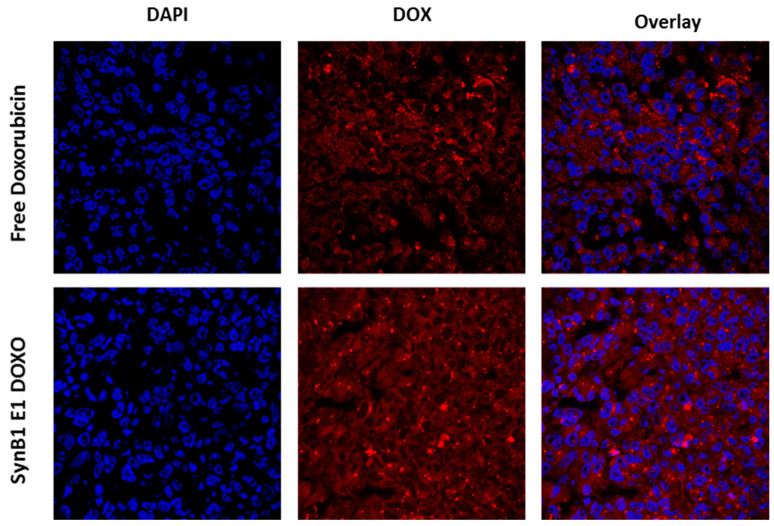
Confocal images of breast cancer tumor sections. Distribution of free doxorubicin and Dox delivered by SynB1-ELP-DOXO. Tumor tissue sections were harvested from tumor bearing nude mice 2 h after intravenous injection of Dox or SynB1-ELP-DOXO. Representative images show Dox fluorescence (red) after staining cell nuclei with DAPI (blue) and viewed using a 20× magnification objective.

To determine intratumoral distribution of dox we have collected tumor tissue and processed it for cryo-microtome sectioning. Confocal images of tumor sections extracted from animals treated with free Dox or SynB1-ELP-DOXO were captured for qualitative localization analysis (Figure 6). Representative images in Figure 6 show similar intratumoral distribution of dox regardless of delivery method. However, some dox aggregates due to the heat stimulated accumulation may be noted in tumors treated with SynB1-ELP-DOXO.

### 2.7. Tumor Reduction by SynB1-ELP-DOXO

**Figure 7:**
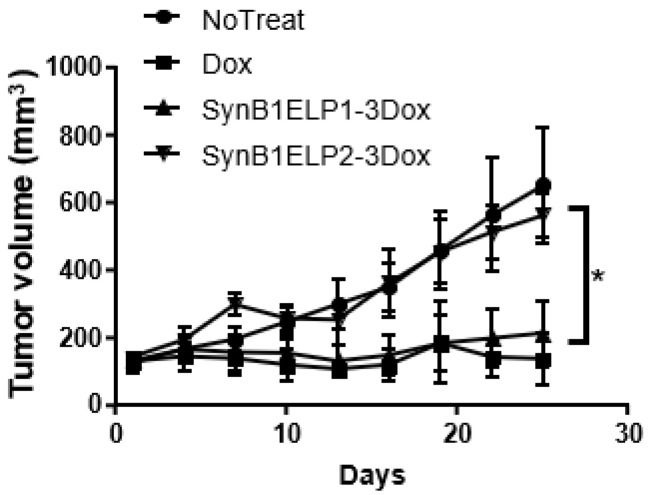
Tumor reduction of SynB1-ELP1-DOXO in MDA-MB-231 xenograft model. SynB1-ELP1-DOXO reduced the growth rate significantly comparing to control and SynB1-ELP2-DOXO treatment (on Day 25). Data were expressed in mean ± SD, *: *p* < 0.05.

The efficacy of SynB1-ELP-DOXO was examined by monitoring the volume of MDA-MB-231 tumor xenografts while administering SynB1-ELP-DOXO. Tumor tissue was heated for 1 h after each injection according to the heating protocol described above (see Methods). Tumor volumes were assessed 3 times weekly by caliper measurement. When treated with SynB1-ELP-DOXO at a dose of 7.5 mg/kg, the MDA-MB-231 tumor volumes were reduced by about 30% over the course of the experiment, while the tumors in PBS treated animals increased about 3-fold in volume. The difference in tumor volume between control and treated tumors was statistically significant beginning on day 20, and the tumors remained significantly smaller for the remainder of the experiment (Figure 7).

While free dox and SynB1-ELP-DOXO tumors were reduced by similar amounts, there is a threefold enhancement in tumor reduction between SynB1-ELP1-DOXO and non-thermally responsive control SynB1-ELP2-DOXO, indicating that our designed ELP drug carrier can be efficiently targeted to the tumor site and effectively inhibit tumor growth in the presence of externally applied, mild hyperthermia.

## 3. Discussion

The anthracycline dox remains among the most potent of anticancer drugs, but its efficacy is limited by irreversible, dose-dependent cardiomyopathy and by emerging clones of tumor cells resistant to its effects. To address these challenges, macromolecular and liposomal encapsulation of drugs, in tandem with the development of drug delivery systems to decrease cardiotoxicity, offer important possible strategies.

Conjugating dox to hydrophilic polymeric carriers improves its solubility, drug delivery and reduces toxicity. As an example, the dox-HPMA copolymer conjugates were clinically evaluated for safety and efficacy [19,20]. Although these HPMA conjugates reduced dox toxicity as compared to free dox, their clinical application has been limited by their variable tumor uptake and fast renal clearance.

The liposomal form of dox (Doxil), currently used to treat several varied types of cancers, reduces cardiotoxicity and enhances systemic drug circulation, but shows concerning side effects, including concentration gradient dependent non-specific release of Dox from the liposomes, which can result in infusion reaction, hypersensitivity and severe dermal toxicity hypersensitivity [21,22,23]. A clinically approved formulation of thermosensitive liposomes, ThermoDox (Celsion Corp., Lawrenceville, NY, USA), has been used in combination with hyperthermia to treat breast cancer and liver tumors [24,25,26]. However, in clinical trial studies of ThermoDox, treatment related neutropenia has been a dose-limiting factor. These liposomes have fast drug release at the tumor site, but still leak a considerable fraction of dox into the plasma, which may account for their toxicity. Such inherent limitations of macromolecular and liposome carriers for dox delivery underscore the advantages of the recently developed ELP system for efficient, targeted dox delivery.

We have shown that biopolymer conjugated drug can be used in thermally targeted treatment of breast cancer in a murine xenograft model. Our results indicate that SynB1-ELP1-DOXO has similar toxicity in tumor reduction when compared to free dox. In addition, the ability of ELP to be thermally targeted reduces the detrimental effects to other healthy tissues that are caused by doxorubicin. The biodistribution study confirmed that the tumor to heart ratio is higher when using SynB1-ELP-DOXO, meaning there is higher doxorubicin delivery to the tumor and less to the cardiac tissue, when compared to free doxorubicin. We do note, however that renal-targeted therapeutics using elastin-like polypeptides are being developed by Bidwell et al. [27]. In these studies, the ELP was modified with a seven-amino acid kidney-targeting peptide (KTP). Biodistribution studies show that this KTP-ELP had a longer plasma half-life than ELP in rat models; similarly, KTP-ELP accumulated in the kidneys at levels fivefold higher than untargeted ELP and showed renal levels 15- to over 150-fold higher than untargeted ELP in other major organs. These findings indicate that ELPs can be used to target different organs, such as the kidneys. Modifying the ELP sequence with different tumor-targeting proteins can permit a similar approach to be applied to metastatic tumors, including brain and kidney tumors.

Tumor reduction previously reported by Moktan et al. [13], showed similar tumor reduction with free dox and SynB1-ELP-DOXO. Due to the difference in animal strain (C57BL/6 vs. nu/nu in present study) we chose to use a different treatment dosage. Regardless, it is confirmed that our thermoresponsive ELP construct can be targeted to the tumor site by local application of hyperthermia and is more efficient than a non-thermally responsive ELP in inhibiting tumor growth. It is important to note that while SynB1-ELP2-DOXO accumulates in the tumor more than free DOXO, because of the different mechanism of action it is much less potent than free DOXO, and therefore it is less effective in reducing the tumor size. It is interesting to note that tumor accumulation of the non-thermoresponsive control SynB1-ELP2-DOXO does not directly translate into efficient tumor reduction. We have previously shown that the thermoresponsive ELP1 accumulates in the tumor much more rapidly than the non-thermoresponsive ELP2. However, the ELP1 construct also remains in the tumor for a prolonged period, while the ELP2 construct not only shows less initial accumulation, but also a much more rapid clearance from the tumor. With tumors thus less exposed to doxorubicin delivered with an ELP2 rather than an ELP1, the less efficient tumor reduction seen with an ELP2 carrier is unsurprising.

A different approach using ELP to deliver dox was used by Mackay et al. [27]. In this study up to 8 dox molecules were conjugated to the ELP macromolecule, resulting in micelle-like nanoparticles formation, with the Dox molecules in the center of the nanoparticles decorated by ELP. Application of these nanoparticles in a C26 murine model of colon carcinoma resulted in nanoparticle accumulation in the tumor and reduced tumor size. These particles accumulated in the tumor due to passive targeting and enhanced permeability and retention effect, which is a phenomenon caused by inherent tumor pathophysiology. In contrast to passive targeting, the ELP drug delivery system described in this report is also actively targeted to the tumor by applying heat as an external stimulus. In addition, using analytical ultracentrifugation, it has been shown that the designed construct does not form nanoparticles at the concentrations used for treatment [28,29,30].

Although dox can be efficiently targeted by the ELP construct and achieve effective inhibition of tumor growth, cancer cell exposed to anthracyclines often develop drug resistance, the major obstacle for anticancer therapy. Since ELP is genetically engineered, it may be easily modified to include a tetrapeptide cleavable linker (GFLG) which is conjugated to a dox molecule without the hydrazone linker used in this study, using simple molecular biology techniques. The GFLG peptide sequence serves as a substrate for lysosomal proteases, and it is cleaved in the lysosomes, releasing doxorubicin into the cell. The released dox molecule will retain two amino acids (L and G) bound to it. This modified form of doxorubicin is not a known substrate for the P-glycoprotein drug pump, which is one well-documented cause of drug resistance. Therefore, ELP-Dox conjugates may not only provide a means of thermally targeted delivery, but they can be designed to overcome inherent or acquired multidrug resistance in hard-to-treat cancers.

## 4. Materials and Methods

### 4.1. Polypeptide Expression and Purification

Polypeptides SynB1-ELP1-(GGC)_3_ and SynB1-ELP2-(GGC)_3_ are designed and synthesized using directional molecular cloning as previously described [13]. The protein was then synthesized by overexpression [31] in *E. coli* BLR (DE3) competent cells (Novagen, Madison, WI, USA) and purified by inverse thermal cycling [32,33]. 

### 4.2. Conjugation of Doxorubicin Derivatives to ELP

Purifed protein SynB1-ELP-(GGC)_3_ at 100 µM concentration was solubilized in elution buffer (50 mM sodium hydrogen phosphate (Na_2_HPO_4_)), pH = 7, with addition of 10-fold molar excess (1 mM) of tris (2-carboxyethyl) phosphine (TCEP) for 30 min at room temperature. Doxorubicin derivative with acid-cleavable (6-maleimidocaproyl) hydrazone linker was synthesized as previously described by Kratz et al. [34] (DOXO-EMCH, CytRx Pharmaceuticals, Freiburg, Germany). Freshly prepared 800 µM DOXO-EMCH was added to the protein solution and left to incubate for another 30 min at room temperature in the dark, followed by O/N incubation at 4 °C, protected from light. The inverse transition cycling procedure was repeated until very little to no free Dox remained in the supernatant. The final protein–Dox sample was collected in PBS, pH 7 and stored in aliquots at −20 °C. Dox levels were determined by spectrophotometry. The absorbance values at 495 and 280 nm were recorded, and the concentration of Dox in the protein–drug sample was calculated by dividing the Dox absorbance at 495 nm by the extinction coefficient of Dox, 9250 M^−1^ cm^−1^. Protein concentration and labeling efficiency was estimated by measuring absorbance at 280 nm and 495 nm. The protein-drug concentration was calculated as described [17].

Drug: Protein conjugate = (Abs 280 nm − (0.713 × Abs 495 nm))/ε_protein_

### 4.3. Cell Lines

MDA-MB 321 human breast cancer cell lines were purchased from ATCC (American Type Culture Collection). Cells were maintained in Dulbecco’s modified Eagle’s minimum essential medium (DMEM) (Corning, ThermoFisher Scientific, Hampton, New Hampshire, NH, USA), supplemented with 10% fetal bovine serum (FBS) (Atlanta Biologicals, Lawrenceville, GA, USA) and 1% Penicillin/Streptomycin antibiotics (HyClone, ThermoFisher Scientific, Hampton, NH, USA). The cells were cultured at 37 °C with 5% CO_2_, and 95% humidity, in exponential growth phase. Every two to three days cells were split using 0.05% Trypsin (HyClone, ThermoFisher Scientific, Hampton, NH, USA).

### 4.4. Animals

Female athymic nu/nu mice were purchased from Envigo at age of 6–8 weeks. All animal experiments were conducted in accordance with the National Institutes for Health Guide for the Care and Use of Laboratory Animals and were approved by the University of Mississippi Medical Center’s Institutional Animal Care and Use Committee.

### 4.5. Tumor Implantation

MDA-MB 231 cells were grown to 80% confluence and harvested with 0.05% trypsin digestion. Cells were washed in PBS, counted using a Coulter cell counter (Z1 Series Particle Counter). For subcutaneous tumor implantation, cells were suspended in a 1:1 ratio with Corning^®^ Matrigel^®^ Matrix (Corning Incorporated Life Sciences, Tewksbury, MA, USA). Nude athymic mice were then anesthetized with isofluorane, and 3 × 10^6^ cells/200 μL/mouse were injected subcutaneously near the fat pad of the fourth mammary gland in the lower left abdomen.

### 4.6. Tumor Reduction Experiments

General performance and survival of the mice were monitored twice weekly, and the size of the tumor xenografts (longitudinal length and transverse width) were measured using an electronic digital caliper, and the measurements were applied in calculating the xenograft volume (π/6 × width^2^ × length). The xenografts were allowed to develop for specific durations of time as described below before further characterization with in vivo imaging inspections and histological analysis. When tumor volumes reached approximately 150 mm^3^, the animals were assigned to one of following four groups at Day 0: PBS (CTRL), free Dox, SynB1-ELP1-DOXO, SynB1-ELP2-DOXO. Subsequently, Day 1 of the study’s treatment phase began. To ensure statistical significance, eight mice were used per group.

### 4.7. Biodistribution and Tumor Accumulation 

After the tumors reached a size of 150 mm^3^ (±10%), the mice were divided into groups of four, and treatments were administered as follows: (1) Control—no treatment (Saline), (2) SynB1-ELP1-DOXO (Tt = 41 °C), (3) SynB1-ELP2-DOXO (Tt = 65 °C), and (4) free Dox, at a concentration of 10 mg/kg doxorubicin equivalent dosage. Applying mild hyperthermia (41 °C) at the tumor site causes ELP1 to form aggregates, substantially pooling it in the tumor microenvironment. Application of mild heat was conducted using a thermal cycling protocol where the heat source is applied for 20 min and withdrawn for 10 min. This heating procedure was performed with the Mettler Laser-System 540^®^ Laser System (Mettler Electronics, Anaheim, CA, USA). This 10 min without heat ensures that ELP-formed aggregates retained in the tumor vasculature will resolubilize and distribute throughout the tumor, exploiting the tumor’s property of leaky vasculature. After the heating cycles, mice were sacrificed at three different time points: 2 h, 8 h, and 24 h. Immediately upon sacrifice, major organs were harvested: tumor, heart, lungs, liver, kidneys, spleen, and brain. From IVIS we estimated the biodistribution of free dox, SynB1-ELP1(DOXO)_3_, and SynB1-ELP2-(DOXO)_3_ by determining the average radiant efficiency of each of the representative images. Tumor accumulation was determined using a similar xenograft implantation protocol. Female athymic nu/nu mice, 6–8 weeks of age, were implanted with 3 × 10^6^ MDA-MB-231 breast cancer cells. When the tumors reached approximately 150 mm^3^, the animals were administered 8 mg/kg of SynB1-ELP-(DOXO)_3_ polypeptides intravenously and the tumors were immediately heated with infrared laser for 2 h. The animals were sacrificed and the tumors were then harvested for tumor tissue slide preparation. Tumor sections were performed by using a cryostat (Leica Biosystems). After MeOH fixation, the slides were stained with DAPI (IHCWorld) to counterstain nuclei. The tumor tissue slides were viewed under a Nikon confocal microscope with 20× objective (Nikon C2+, Nikon Instruments Inc., Melville, NY, USA) and each image was captured with Ni+ elements and processed by ImageJ (National Institute of Health, Bethesda, MD, USA) software.

## 5. Conclusions

Thermally targeted ELP-based biopolymers enhance tumor targeting and uptake of anticancer drugs and minimize free drug toxicity in healthy tissues. Efficiency of uptake in tumor tissue and cancer cells of ELP-delivered dox in tumor was further enhanced by the addition of a cell penetrating peptide. Using ELP for delivery of dox significantly reduces accumulation of doxorubicin in the heart, therefore minimizing potential heart damage Furthermore, conjugation of dox to ELP, in combination with applied hyperthermia, extended the drug half-life and increased tumor uptake, indicating that ELP-based drug carriers have great potential for clinical application.

## Data Availability

The data presented in this study are available in the article and on request from the corresponding author.
